# ECG-derived Poincaré plot graphical metrics for differentiating axillary lymph node metastasis in breast cancer

**DOI:** 10.3389/fphys.2026.1795471

**Published:** 2026-05-20

**Authors:** Nannan Fang, Aoqi Zhang, Zhijing Song, Zongyu Xie, Bo Shi, Zhiwen Wang

**Affiliations:** 1 Anhui Key Laboratory of Digital Medicine and Intelligent Health, School of Medical Imaging, Bengbu Medical University, Bengbu, China; 2 Department of Radiology, The First Affiliated Hospital of Bengbu Medical University, Bengbu, Anhui, China

**Keywords:** autonomic nervous system, axillary lymph node metastasis, breast cancer, heart rate variability, Poincaré plot

## Abstract

**Background:**

Axillary lymph node metastasis (ALNM) status serves as a cornerstone for clinical decision-making in personalized breast cancer (BC) management. Although autonomic nervous system (ANS) dysfunction correlates with tumor progression, its applicability for capturing BC-ALNM-specific ANS signatures remains exploratory. This proof-of-concept study aimed to investigate whether Poincaré plot analysis of short-duration electrocardiograms could potentially identify ANS signals associated with ALNM more effectively than conventional heart rate variability (HRV) metrics.

**Methods:**

Resting electrocardiograph data were collected from 218 pathologically confirmed BC patients at the First Affiliated Hospital of Bengbu Medical University (142 ALNM-positive, 76 ALNM-negative). Conventional HRV metrics and Poincaré plot-derived spatial metrics were compared using raw and detrended data. Beyond univariable comparisons evaluated by Cohen’s d effect sizes with 95% confidence intervals (CIs), multivariable Analysis of Covariance (ANCOVA) adjusting for clinicopathological confounders were employed to ensure model robustness. Discriminative performance was evaluated using Receiver Operating Characteristic (ROC) analysis. To assess the potential for overfitting, a 10-fold cross-validation procedure was implemented for the ROC analysis.

**Results:**

Conventional metrics showed no significant intergroup differences or predictive value (AUC = 0.532). Univariable analysis revealed differences in the detrended Area Index (AI) (*p* = 0.006, Cohen’s *d* = 0.40, 95% CI: 0.12-0.68), and raw grid distribution metrics (GDR, GDE) showed significant differences between groups. However, after strict multivariable adjustment, only the detrended AI remained a significant independent predictor (adjusted *p* = 0.024). The original AUC of the detrended AI was 0.612, and the 10-fold cross-validated AUC was 0.607, indicating robustness against overfitting.

**Conclusions:**

In this exploratory study, Poincaré plot analysis, specifically the detrended AI, suggests a potential methodological advantage for characterizing ALNM-associated autonomic spatial asymmetry independent of general tumor burden. However, given the modest discriminative ability and single-center design, these findings must be interpreted with caution. These non-invasive metrics may offer a novel pathophysiological perspective but require future large-scale, multi-center prospective studies to overcome current limitations and validate their clinical utility.

## 1 Introduction

Axillary lymph node metastasis (ALNM) status is a cornerstone for clinical decision-making in personalized breast cancer (BC) management, directly influencing therapeutic strategies and prognostic evaluation (Carter et al., 1989; Beenken et al., 2003; Soerjomataram et al., 2008). Currently, ALNM detection predominantly relies on invasive procedures such as sentinel lymph node biopsy and imaging modalities (Caudle et al., 2014; Gong et al., 2022). However, these approaches are often costly, carry inherent risks, and can yield false-negative results (Bedrosian et al., 2003; Wojcinski et al., 2012; Park et al., 2014). Consequently, there is an urgent clinical need for the development of cost-effective, non-invasive auxiliary tools to improve ALNM assessment.

Accumulating evidence suggests a correlation between autonomic nervous system (ANS) dysfunction and tumor progression (Sloan et al., 2010; Magnon et al., 2013; Cole et al., 2015). The ANS modulates critical processes within the tumor microenvironment, which include inflammation (Armaiz-Pena et al., 2015; Mohammadpour et al., 2021), angiogenesis, and the metastatic cascade (Tracey, 2007; Cole et al., 2015), primarily through the sympathetic-adrenal axis and cholinergic anti-inflammatory pathway. Consequently, patterns of ANS activity may serve as potential indicators of tumor aggressiveness and metastatic potential. Heart rate variability (HRV), defined as the fluctuation in time intervals between consecutive sinus beats (R-R intervals, RRIs) (Zhou et al., 2016), provides a well-established, non-invasive quantitative metric for assessing cardiac autonomic regulation (Xhyheri et al., 2012). As such, HRV analysis has emerged as a prevalent tool for investigating ANS activity patterns in various malignancies (Vigier et al., 2021; Wu et al., 2021).

Although direct comparisons of HRV specifically between ALNM-positive and ALNM-negative patients in early-stage breast cancer remain scarce, emerging evidence from related oncology fields suggests a plausible pathophysiological link. Supporting this possibility, preclinical models have demonstrated that sympathetic nervous system hyperactivation actively remodels the lymphatic vasculature, structurally and functionally facilitating tumor cell dissemination into lymph nodes (Le et al., 2016). Complementing this, clinical studies have shown that patients with advanced-stage breast cancer exhibit significantly reduced HRV (including non-linear parameters) compared to those with early-stage or benign disease (Bettermann et al., 2001), with more recent work further associating reduced HRV complexity with advanced disease stages and poorer prognosis (Giese-Davis et al., 2015). Similarly, investigations in other solid malignancies have identified preoperative short-term HRV as a potential independent predictor of lymph node metastasis, where diminished HRV correlates with lymphatic invasion and nodal involvement (Wang et al., 2021). Nevertheless, whether such distinct autonomic signatures can be effectively captured to differentiate ALNM status early in the breast cancer course remains systematically unvalidated, representing a critical knowledge gap that the present study seeks to address.

Furthermore, it is well-established that cardiac autonomic tone is profoundly influenced by baseline physiological factors, particularly age and hormonal status (Kuo et al., 1999). For instance, endogenous estrogens exert a modulating effect on the ANS by enhancing parasympathetic (vagal) tone; consequently, the menopausal transition is typically accompanied by a global decline in HRV and a shift toward sympathetic dominance (Neves et al., 2007). Given these physiological realities, when investigating disease-specific autonomic signatures in female breast cancer cohorts, it is imperative to rigorously acknowledge and account for inherent demographic and hormonal variations to ensure that the observed HRV alterations are genuinely driven by tumor pathology rather than intrinsic endocrine shifts.

Conventional HRV analysis primarily utilizes time-domain, frequency-domain, and traditional non-linear metrics to quantify aspects of RRI variability, spectral power distribution, and overall complexity. While informative about global sympatho-vagal balance, these conventional metrics face significant challenges in capturing the complex, non-stationary dynamics characteristic of short-term electrocardiograph (ECG) recordings (Huikuri et al., 2000; Billman, 2013; Gu et al., 2023). Moreover, global linear metrics are largely sensitive to non-specific depression of autonomic tone driven by general metabolic exhaustion or total tumor burden (Kim et al., 2010; Arab et al., 2016), yet they may have limited ability to specifically capture the highly dynamic neuro-inflammatory crosstalk characteristic of the metastatic cascade. This limitation raises questions about their ability to fully characterize the intricate features of cardiac ANS activity under specific pathological conditions like BC-associated ALNM.

Poincaré plot analysis offers an intuitive graphical non-linear method for HRV assessment by constructing a scatter plot of consecutive RRIs (Brennan et al., 2001; Nayak et al., 2018; Satti et al., 2019). This approach generates graphical metrics (e.g., ellipse fitting metrics (Kamen et al., 1996), asymmetry metrics (Guzik et al., 2006; Porta et al., 2008; Karmakar et al., 2015; Yan et al., 2017), and gridding metrics (Yan et al., 2019) that aim to characterize the dynamic properties, distributional heterogeneity, and complexity of RRIs in a more fine-grained manner than conventional methods. Theoretically, this graphical representation holds promise for more effectively identifying subtle ANS dysregulation patterns associated with complex pathophysiological states (Yan et al., 2021).

Therefore, given the established link between ANS dysfunction and cancer progression, this exploratory, proof-of-concept study aimed to systematically evaluate whether Poincaré plot-derived metrics could effectively capture BC-ALNM-specific ANS signatures compared to conventional HRV metrics. Short-term resting ECG signals were collected from BC patients and a comprehensive panel of standard and graphical Poincaré metrics was calculated a comprehensive panel of standard and graphical Poincaré metrics. To rigorously isolate the metastasis-specific autonomic signal from baseline physiological noise and general tumor burden, metrics were evaluated using both raw and detrended RRIs, alongside stringent multivariable adjustments. Ultimately, this study sought to determine whether spatial Poincaré plot analysis can offer a novel pathophysiological perspective and serve as a potential non-invasive adjunctive tool for optimizing tumor assessment.

## 2 Methods

### 2.1 Subjects

This study was conducted at The First Affiliated Hospital of Bengbu Medical University between December 2023 and September 2024. The study population comprised female patients diagnosed with invasive BC to investigate their resting ECG characteristics. A total of 249 patients initially diagnosed with invasive BC via core needle biopsy were screened for enrollment. To ensure data reliability, completeness, and cohort homogeneity, explicit exclusion criteria were applied, which are absence of critical clinical or pathological data (23 cases), diagnosis of ductal carcinoma *in situ* (1 case), poor ECG signal quality precluding reliable analysis due to severe arrhythmia (6 cases), and recurrent BC (1 case). Consequently, 218 eligible patients were enrolled. Based on the key clinical metric of ALN status, the cohort was classified into ALNM-positive (142 cases) and ALNM-negative (76 cases). The study protocol was approved by the Clinical Medical Research Ethics Committee of The First Affiliated Hospital of Bengbu Medical University (Bengbu, Anhui, China; Approval number: 2025155) and complied with the ethical principles of the Declaration of Helsinki. All participants provided written informed consent after being fully informed of the research objectives, procedures, potential risks, and benefits.

Essential clinical information, including patient age, body mass index (BMI), and menopausal status, was collected and documented through review and extraction from the hospital’s electronic medical record system. ALN pathological status was definitively based on surgical specimens obtained during either sentinel lymph node biopsy (SLNB) or axillary lymph node dissection (ALND). Pathological diagnosis was independently performed and confirmed by two experienced pathologists to maximize diagnostic accuracy. Lymph node metastasis positivity was defined according to standardized pathological criteria as follows: (i) Macrometastasis: Largest metastatic deposit > 2.0 mm; (ii) Micrometastasis: Largest metastatic deposit > 0.2 mm and ≤ 2.0 mm, or tumor cell cluster > 200 cells in a single histological section. Deposits ≤ 0.2 mm (or tumor cell cluster ≤ 200 cells) were classified as isolated tumor cells (ITCs) and considered ALNM-negative (Singletary and Connolly, 2006; Amin et al., 2017).

### 2.2 ECG acquisition

**Figure 1:**
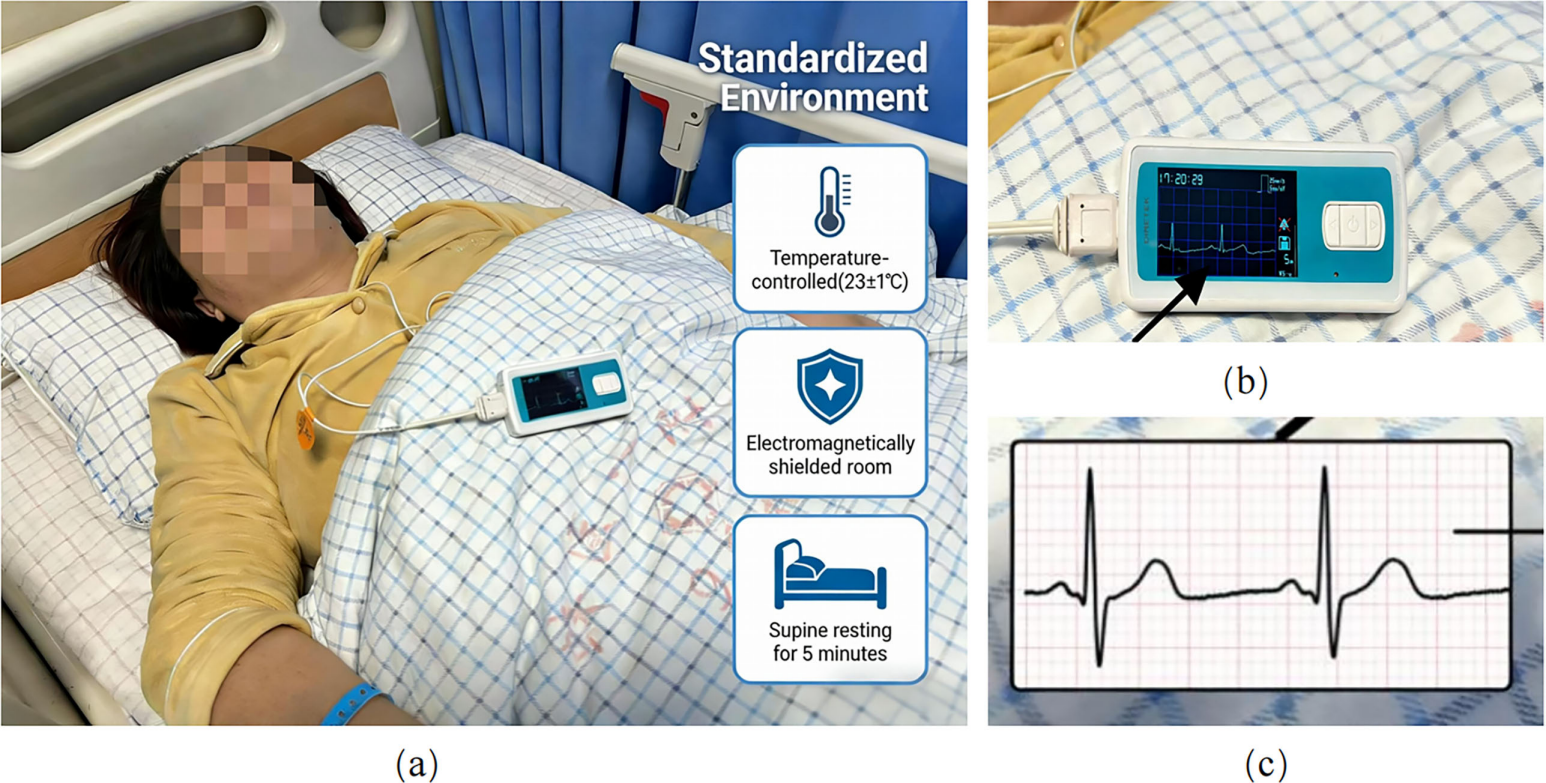
Standardized environment, equipment, and a representative raw ECG trace for HRV data acquisition. **(A)** shows the standardized acquisition environment: the patient rests quietly in the supine position for 5-minutes inside a temperature-controlled (23 ± 1°C) and electromagnetically shielded room, which minimizes external interference and ensures that the recordings reflect the true baseline autonomic state. **(B)** shows a single-lead micro-ECG recoder (sampling frequency 400 Hz, bandpass filter 0.6–40 Hz) used to continuously collect resting-state ECG data from the V6 lead position. **(C)** shows a representative raw ECG waveform, suitable for subsequent R-wave peak detection and heart rate variability analysis.

Resting ECG data was acquired following a standardized protocol to minimize the impact of confounding variables. A single-lead micro-ECG recorder was used to collect continuous 5-minute resting-state ECG data from each patient at the V6 lead position. The V6 lead was chosen based on its established ability to yield a stable, high-amplitude R-wave signal, as well as its comparatively lower susceptibility to motion artifacts and respiratory interference than other leads (Kligfield et al., 2007; García-Niebla et al., 2009). Data acquisition was performed in a temperature-controlled (23 ± 1°C) and electromagnetically shielded room. Patients rested quietly in a supine position. The recorder used a sampling frequency of 400 Hz and a bandpass filter of 0.6–40 Hz to capture the main ECG activity (**Figure 1**). It should be noted that this high sampling frequency provides a fine time resolution of 2.5 ms. According to standard guidelines for HRV analysis, a sampling frequency of 250–500 Hz is optimal, as it significantly reduces quantization error and the probability of inaccurate R-wave peak detection, making it the recommended range for clinical and research applications (Task Force of the European Society of Cardiology and the North American Society of Pacing and Electrophysiology, 1996). Throughout the data collection process, the subject was required to remain still and maintain steady breathing to obtain high-quality signals reflecting the baseline physiological state.

### 2.3 Automated RRI extraction and detrending

Following ECG acquisition, this study established an automated, high-fidelity process for extracting RRIs and subsequent preprocessing. This process aimed to accurately identify valid cardiac beat fiducial points from the raw ECG signal, apply rigorous quality control, and perform detrending, establishing a reliable data foundation for the in-depth evaluation of autonomic nervous function status in BC patients.

**Figure 2:**
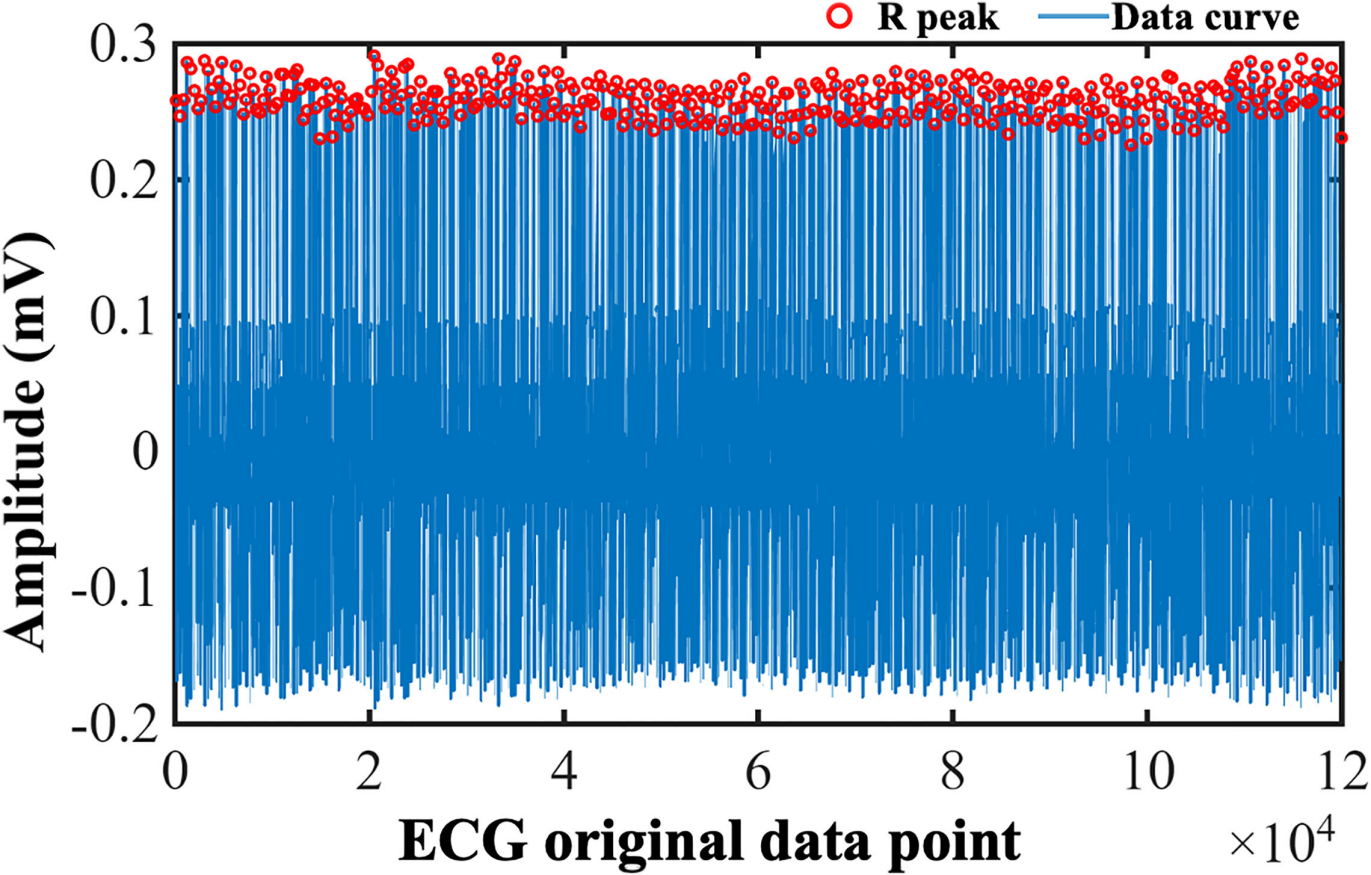
Example of detrended ECG signals from a representative patient. The vertical axis represents signal amplitude (mV), and the horizontal axis denotes raw data points. R-peaks marked in red circle. The curve shows periodic fluctuations reflecting cardiac electrical activities.

**Figure 3:**
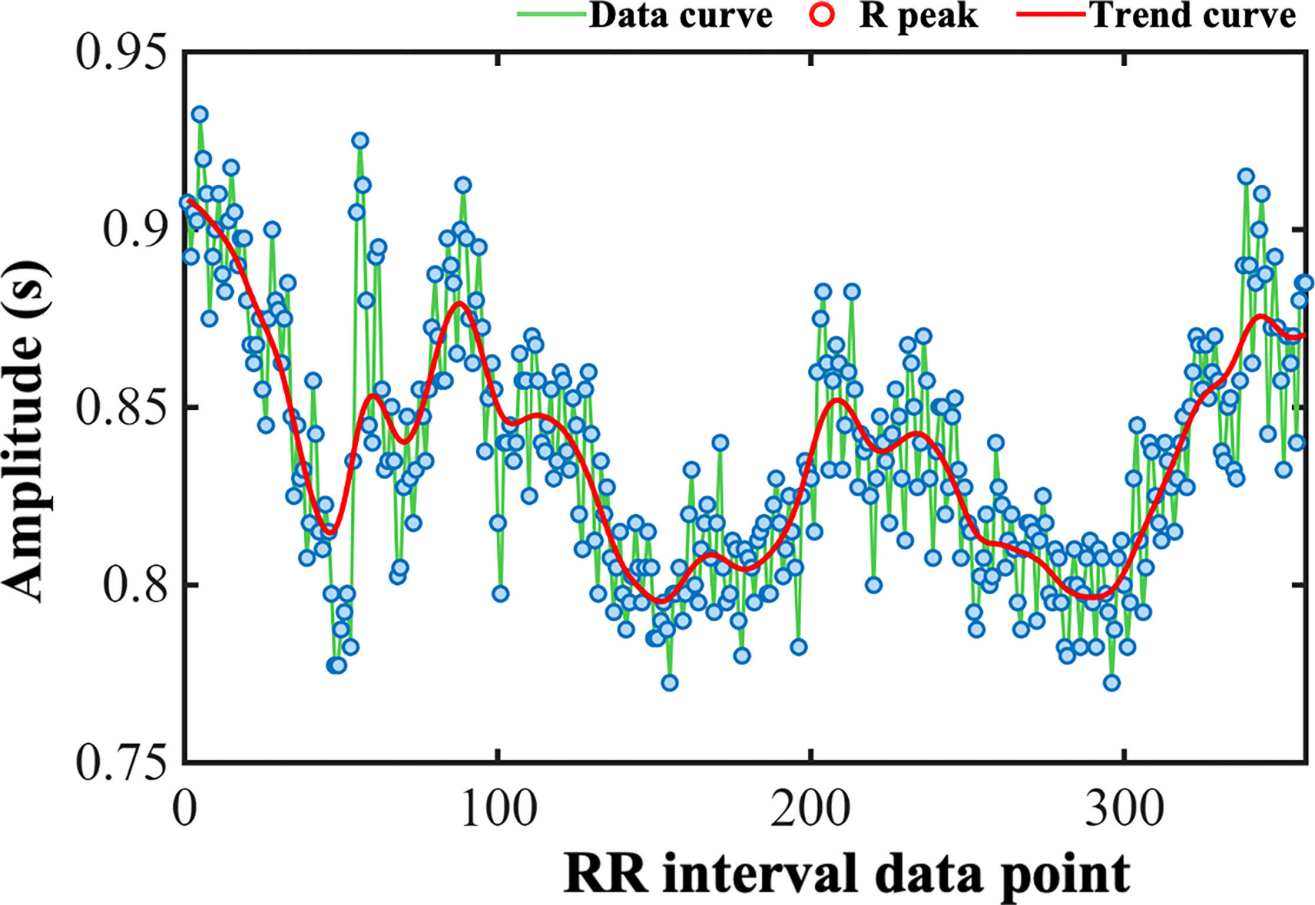
Fluctuations in the raw RRI series from a representative patient. Blue dots represent individual intervals, and connecting lines illustrate the temporal variation of the sequence, and the red line denotes the trend curve. The x-axis shows RRI extraction data points, while the y-axis indicates amplitude (unit: s).

RRI extraction employed an adaptive QRS complex detection algorithm based on Pan-Tompkins method (Pan and Tompkins, 1985). Initial ECG voltage data (representative morphology shown in **Figure 2**) underwent preprocessing with a 5–15 Hz bandpass Butterworth filter to effectively suppress baseline wander and high-frequency electromyographic noise. Subsequently, filtered signals were subjected to a five-point differencing operation to enhance the steep slope information of the QRS complex and squaring to further improve the signal-to-noise ratio of the target waveform. To consolidate local signal energy, smoothing was applied using a 150-millisecond moving average window. An advanced peak detection algorithm, integrating adaptive dynamic thresholds (to distinguish signal peaks from noise), a minimum peak interval constraint (0.2 seconds), a maximum T-wave identification interval (0.36 seconds), and a refractory period setting (0.2 seconds), was used to identify candidate R-peak locations. To ensure positional accuracy, the algorithm performed a local maximum search within a ±150 millisecond window around each candidate point in the original filtered signal to definitively confirm the valid R-peak apex. The time interval between consecutive valid R-peak apices constituted the raw RRI series, visualized in **Figure 3**, where the y-axis represents the RRI values, blue dots indicate individual intervals, and connecting lines illustrate the temporal variation of the sequence.

**Figure 4:**
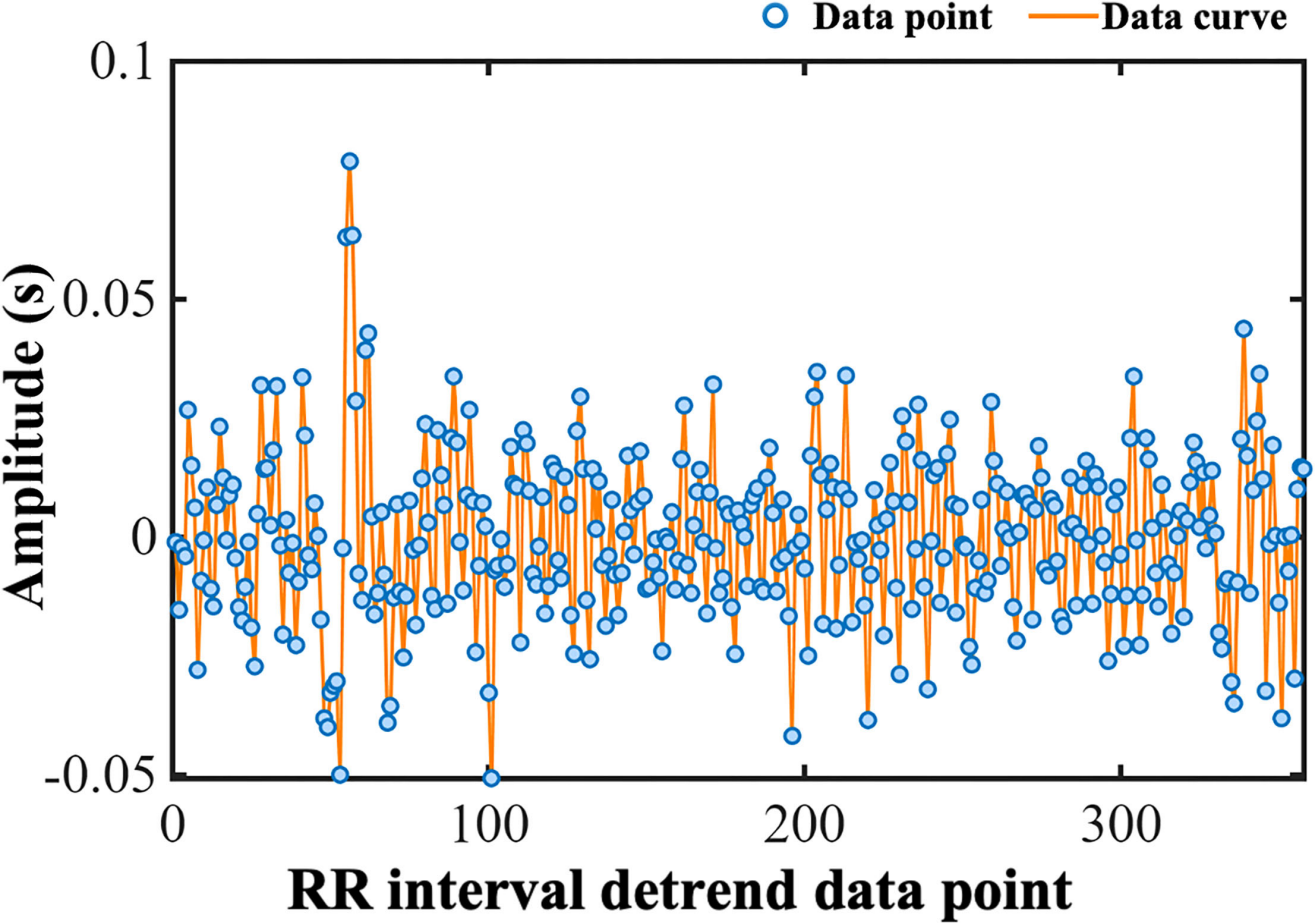
Fluctuations in the detrended RRI series in a representative patient. Blue dots represent individual intervals, and connecting lines illustrate the temporal variation of the sequence, The x-axis denotes RRI detrend data point, and the y-axis shows amplitude (unit: s). This figure illustrates fluctuations in cardiac cycle intervals after detrending, supporting heart rate variability analysis.

Given that raw RRIs commonly contain outliers caused by noise, artifacts, and ectopic beats, stringent data quality control was critical. A specifically designed RRI filtering algorithm was implemented for meticulous screening, where intervals less than 0.3 seconds or greater than 2.0 seconds were unconditionally discarded as physiologically implausible at rest. If the absolute relative change rate between adjacent RRIs exceeded 20%, both the data point and its immediate neighboring points were flagged as potential abnormal clusters. To probe and effectively eliminate the potential influence of non-stationary low-frequency trend components within the RRI series on short-term autonomic fluctuation analysis, Smoothness Priors detrending (Tarvainen et al., 2002) was applied (corresponding to the red trend line in **Figure 3**; detrended effect shown in **Figure 4**). This algorithm fits a highly smooth trend component and subtracts it from the original valid RRI series. The algorithm settings used a smoothing strength parameter λ=500 and a difference order of 2. The resulting detrended RRI series minimized extraneous low-frequency drifts, primarily reflecting autonomic-mediated short-term (high-frequency) heart rate modulation.

### 2.4 Conventional HRV metrics

Using the precisely extracted and preprocessed RRI series, conventional HRV metrics were calculated across multiple dimensions (Tarvainen et al., 2014), including time-domain, frequency-domain, non-linear analysis, and Poincaré plot graphical metrics. This comprehensive approach provided a multi-level assessment of cardiac autonomic nervous activity.

Time-domain analysis evaluated overall HRV characteristics using core SDNN (standard deviation of NN intervals) and RMSSD (root mean square of successive differences) metrics. SDNN was calculated as the standard deviation of the entire RRI series, which reflected the overall strength of autonomic tone. The decreased SDNN is widely recognized as significantly associated with increased cardiovascular risk (Wu et al., 2021). RMSSD was calculated as the root mean square of differences between adjacent valid RRIs, specifically characterizing rapid vagal modulation of heart rate and serving as a sensitive marker of parasympathetic activity. These metrics are considered gold standards in HRV assessment, with standardized calculation formulas (Liu et al., 2023).

Frequency-domain analysis focused on spectral characteristics of heart rate oscillations. Power spectral density (PSD) estimation was performed using Welch’s periodogram method. The RRI series was resampled at 4 Hz to obtain a uniformly sampled time series. The resampled data was segmented using a 256-point Hamming window with 50% overlap. Fast Fourier Transform (1024 points) was used to compute the power spectrum (Brennan et al., 2001). Key frequency band power integrals included LF, HF and LF/HF Ratio. LF (Low Frequency Power, 0.04-0.15 Hz) reflects combined sympathetic and vagal activity, associated with regulatory mechanisms like baroreflex activity (Rahman et al., 2011). HF (High Frequency Power, 0.15-0.40 Hz) primarily represents vagal tone, its power modulated significantly by respiratory rhythm, marking rapid parasympathetic modulation. The LF/HF ratio was calculated as the ratio of LF power to HF power, serving as a non-specific marker of autonomic modulation (Shaffer and Ginsberg, 2017).

Non-linear analysis quantified the complexity and unpredictability of the RRI series, revealing intrinsic dynamic properties. Approximate Entropy (ApEn) quantifies time series regularity, such that lower values denote greater predictability. Sample Entropy (SampEn) is a refinement of ApEn, reducing its bias due to data length dependency and offering greater statistical robustness. Reduced SampEn also indicates decreased flexibility in autonomic modulation or loss of system complexity, closely associated with autonomic rigidity found in conditions like aging and diabetic neuropathy (Beckers et al., 2006; Shi et al., 2019).

### 2.5 Quantification of Poincaré plot graphical metrics

The novel Poincaré plot quantification method represents a core contribution of this study. Building upon the conventional Poincaré scatter plot (Kamen et al., 1996), where RR(i) is plotted against RR(i+1) to visualize correlations between consecutive intervals, this study applied three Poincaré plot graphical metrics for analysis.

**Figure 5:**
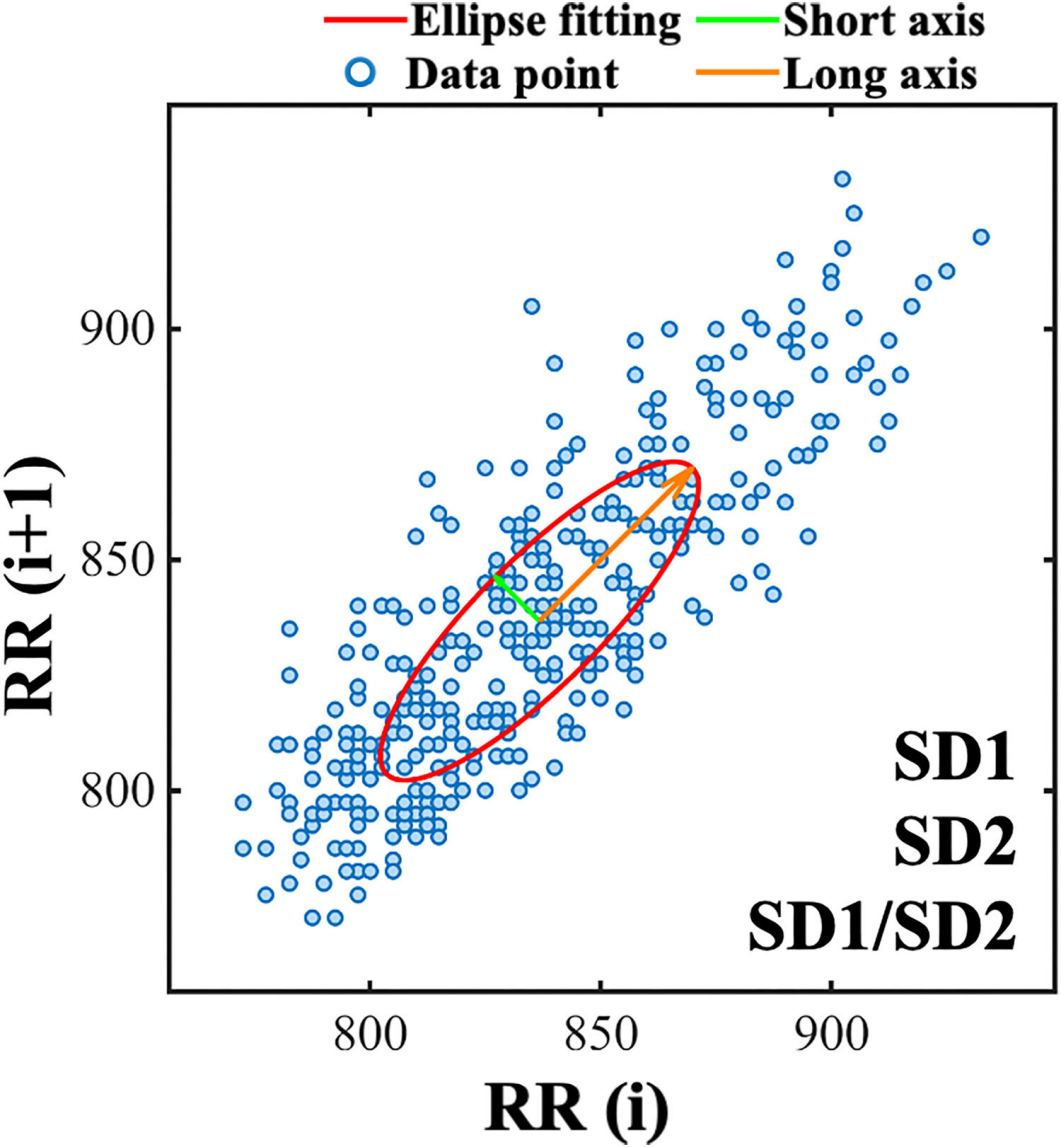
Poincaré plot of RRIs with ellipse fitting from a representative patient. Blue dots represent RRIs data points (RR(i) vs. RR(i+1)). The red curve shows ellipse fitting, with orange/green lines denoting long/short axes. SD1, SD2, and SD1/SD2 quantify heart rate variability features.

Elliptical fitting geometric metrics are shown in **Figure 5**. An elliptical fit (red ellipse) was applied to the Poincaré scatter plot to extract key morphological metrics. SD1 (Semi-minor axis, green line) represents a specific value of the standard deviation of the differences between successive RRIs, quantifying instantaneous (beat-to-beat) HRV predominantly influenced by immediate vagal modulation. SD2 (Semi-major axis, orange line) represents a function of the variance of RR(i) adjusted by SD1, quantifying long-term HRV reflecting slower sympathetic modulation. SD1/SD2 serves as an index of autonomic balance; a reduced ratio suggests relative sympathetic dominance. This analysis provides complementary information to conventional time-domain metrics by capturing sequential dynamics (Brennan et al., 2002).

**Figure 6:**
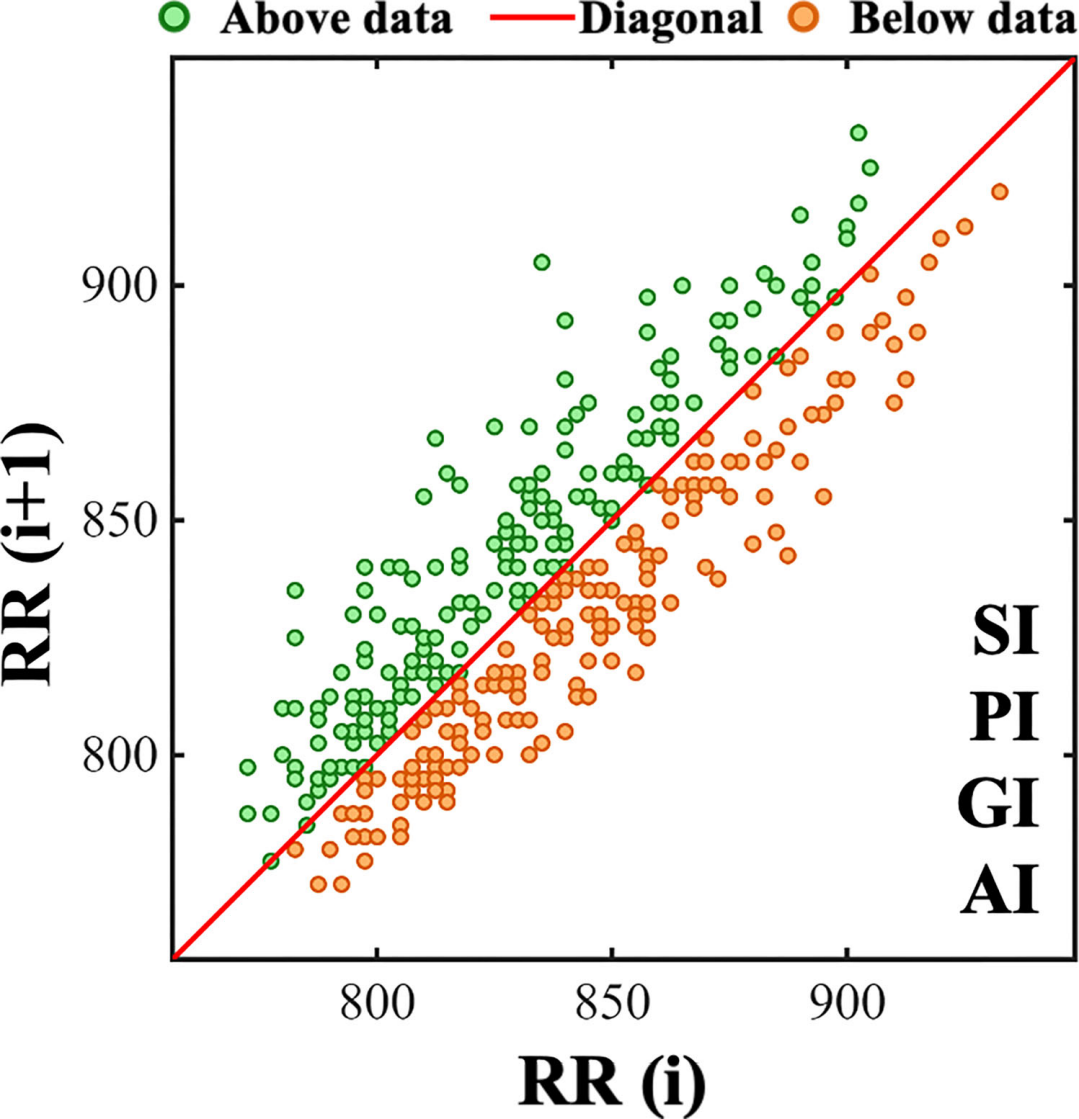
Poincaré plot of RRIs from a representative patient. The x - axis and y - axis represent RR(i) and RR(i + 1), respectively. The red diagonal marks identical consecutive intervals. Above data represents the acceleration quadrant (RR(i+1) < RR(i), green area). Below data represents the deceleration quadrant (RR(i+1) > RR(i), orange area). SI, PI, GI, AI are indices for heart rate variability analysis.

Heart rate asymmetry metrics were shown in **Figure 6**. A red 45° line was introduced to divide the Poincaré plot into an acceleration quadrant (RR(i+1) < RR(i), green area) and a deceleration quadrant (RR(i+1) > RR(i), orange area). Four key metrics were systematically defined to quantify spatial distribution imbalances between cardiac acceleration and deceleration regulation. Porta’s index (PI) represents the absolute difference between the proportion of points within the accelerating quadrant and the 50% theoretical symmetry baseline, quantifying asymmetry in the occurrence frequency of acceleration versus deceleration events. Guzik’s index (GI) is the absolute difference between the ratio of the sum of vertical distances from all accelerating points to the identity line to the sum of distances from all points and 50%, reflecting asymmetry in the magnitude of fluctuations during acceleration and deceleration processes. Slope index (SI) denotes the absolute difference between the ratio of the sum of the absolute phase angles of accelerating points to the sum of the absolute phase angles of all points and 50%, capturing asymmetry in the instantaneous rate of change during these processes. The AI constitutes the absolute difference between the proportion of the area of the accelerating sector to the total area of the Poincaré plot and 50%, characterizing the overall spatial area asymmetry between acceleration and deceleration processes. All metrics are presented as |X-50|, where X is the raw proportion index value. Their magnitude directly reflects the degree of deviation from symmetry; a value of zero indicates perfect symmetry. This approach addresses the implicit symmetry assumption in conventional HRV analysis (Karmakar et al., 2012).

**Figure 7:**
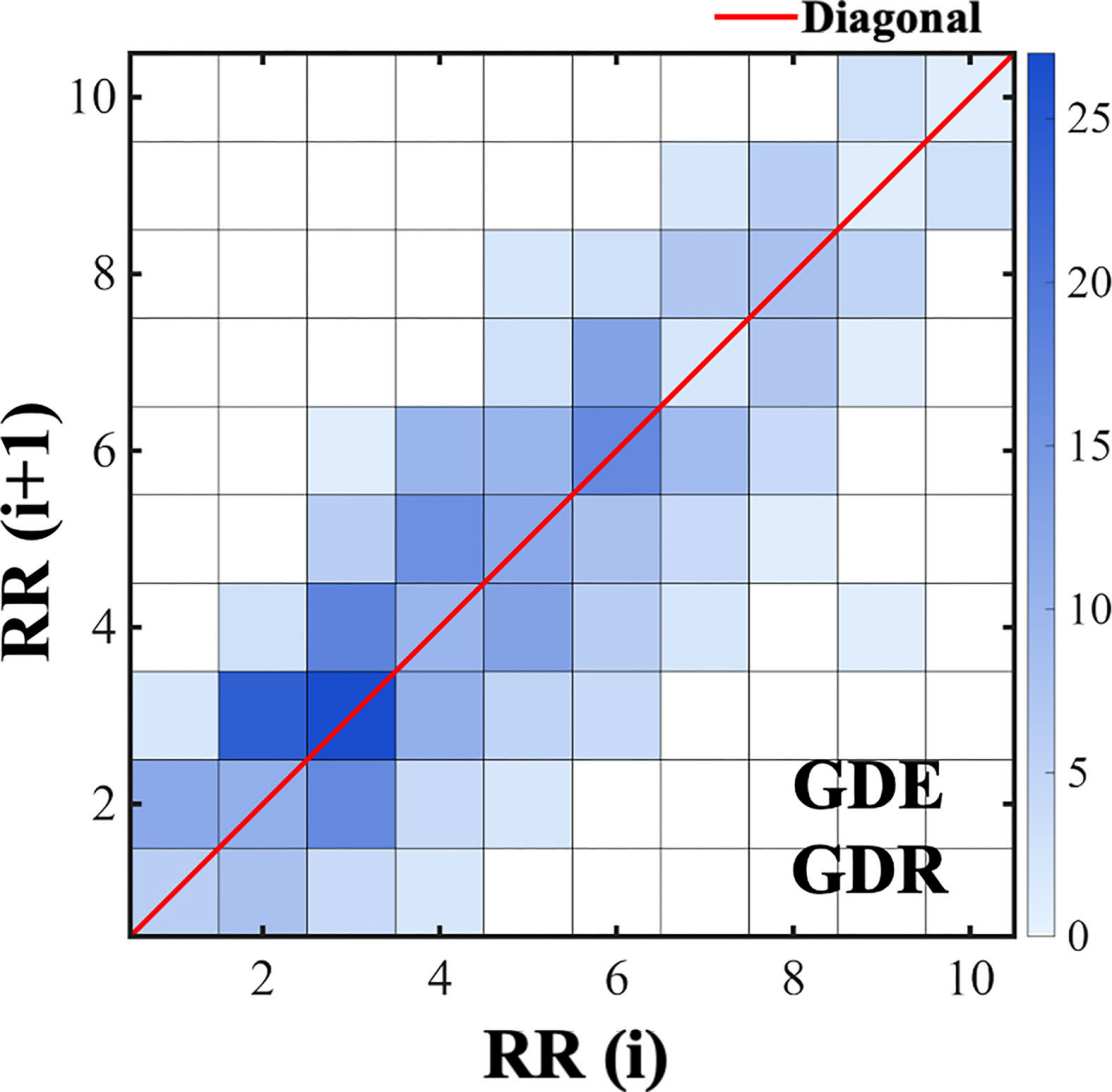
Grid - based RRI distribution plot. The x - axis and y - axis represent RR(i) and RR(i + 1), respectively. Color intensity reflects the frequency of interval occurrences within each grid, with the right - side color bar indicating count values (0–25). The red diagonal marks identical consecutive intervals.

Grid distribution metrics are shown in **Figure 7**. To analyze the microstructure and complexity of point dispersion within the Poincaré plot, the plot plane was discretized into an n × n grid (n dynamically selected between 1 and 200), and two metrics were computed (Yan et al., 2019). Importantly, the choice of grid resolution can significantly impact the stability of spatial distribution metrics. Therefore, rather than relying on a single, arbitrary grid resolution, our algorithm inherently incorporated a sensitivity analysis approach. Specifically, the metrics were calculated across a dynamic, continuous range of grid resolutions from n = 100 to n = 200. The final index values for each patient were derived by averaging the results across this entire range. This multi-resolution averaging approach ensures that the derived GDR and GDE metrics are highly robust, stable, and not spuriously driven by the selection of a specific grid dimension.

GDR represents the proportion of grids containing at least one data point relative to the total number of grids. This metric measures the spatial dispersion of points; a lower GDR value suggests more concentrated rhythmic patterns and weaker randomness. GDE quantifies the randomness and complexity of the spatial point distribution based on the probability of data point occurrence within each grid cell. Higher entropy values indicate more complex and unpredictable distribution patterns. **Figure 7** illustrates point density using color intensity. This approach effectively addresses the limitation of conventional Poincaré analysis being insensitive to local density variations.

### 2.6 Statistical analysis

Following the successful computation of multidimensional quantitative results, encompassing both conventional HRV metrics and the novel Poincaré plot graphical metrics, rigorous statistical assessment of group differences was performed. This aimed to systematically reveal significant biological differences between the group with intact ALN and the group with metastasis. The statistical analysis employed a non-parametric framework, strictly adhering to the principle of combining hypothesis testing with effect size estimation to ensure result robustness, statistical significance, and interpretability of clinical relevance.

The structured dataset containing all calculated metrics for all subjects was imported. This dataset included time-domain, frequency-domain, non-linear, and Poincaré plot graphical metrics. Based on the established clinical grouping labels (ALNM-negative vs. ALNM-positive), the metric data from all 218 patients were strictly partitioned into two independent comparison groups. Normality of continuous variables was assessed using the Shapiro-Wilk test. Normally distributed continuous data are presented as mean ± standard deviation, while non-normally distributed continuous data are expressed as median (first quartile, third quartile). Categorical data are reported as counts (percentages). Group comparisons for continuous variables used the Student’s *t*-test (normally distributed) or Mann-Whitney *U* test (non-normally distributed). The chi-square test was used for categorical variables. The significance threshold (alpha) was set at 0.05. An uncorrected p-value less than 0.05 led to rejection of the null hypothesis, indicating a statistically significant difference for that metric between groups. To move beyond a simple statistical significance binary decision (*p*-value) and assess the practical biological or clinical relevance of detected differences, Cohen’s *d* was calculated concurrently. As a scale-free measure, Cohen’s *d* facilitates objective comparison of effect size magnitudes across different metrics. According to Cohen’s convention, |*d*| > 0.2 indicates a small effect size, |*d*| > 0.5 indicates a medium effect size, and |*d*| > 0.8 indicates a large effect size, directly reflecting the strength of the observed group differences.

Given that the Poincaré plot metrics are geometrically derived from the same spatial distribution and are therefore inherently highly correlated, conventional multiple comparison correction methods (e.g., false discovery rate, Bonferroni) are considered overly conservative for such non-independent variables. These methods may increase type II error (false negative) risk and obscure genuine physiological signals. Consistent with the exploratory nature of this study aimed at identifying novel graphical HRV biomarkers associated with axillary lymph node metastasis, uncorrected *p*-values are primarily reported. To ensure statistical reliability and to provide a more precise quantification of effect sizes, Cohen’s *d* effect sizes with 95% confidence intervals (CI) were calculated for the univariate comparisons.

To further evaluate whether the observed differences in Poincaré plot metrics were independent of potential confounding factors, an analysis of covariance (ANCOVA) was performed. Covariates included age, menopausal status (as a surrogate for systemic estrogen levels), progesterone receptor (PR) expression, and Ki-67 index (as indicators of tumor endocrine subtype and proliferative activity). The ANCOVA model was constructed with group (ALNM-positive vs. ALNM-negative) as the fixed factor and the aforementioned variables as covariates. Adjusted *p* values < 0.05 were considered statistically significant. Partial η² was calculated as a measure of effect size to quantify the proportion of variance explained by group membership after controlling for covariates.

Receiver operating characteristic (ROC) curve analysis was conducted to evaluate the discriminative performance of the Poincaré plot metrics for identifying ALNM. To comprehensively assess methodological superiority, the predictive capability of the novel detrended AI was visually and statistically compared against both conventional HRV metrics (e.g., SDNN) and non-linear HRV metrics (e.g., ApEn). The area under the curve (AUC) was calculated. To assess the potential for overfitting and to evaluate the generalizability of the predictive model, a 10-fold cross-validation procedure was then implemented for the detrended AI. The dataset was randomly partitioned into 10 approximately equal subsets; in each iteration, 9 subsets were used for training and the remaining 1 subset for validation, and this process was repeated 10 times. The cross-validated AUC was calculated by averaging the performance metrics across all folds. This approach provides a more conservative estimate of the model’s discriminative ability and helps ensure that the observed predictive performance is not attributable to overfitting to the specific cohort.

## 3 Results

The study cohort comprised 218 patients. **Table 1** shows the demographic characteristics of the patients, who had a mean age of 50.6 ± 10.1 years and a mean BMI of 25.0 ± 3.4 kg/m2. Based on histopathological findings, patients were classified into ALNM-positive (142 patients, 65.1%) and ALNM-negative (76 patients, 34.9%) groups. The ALNM-positive group had a mean age of 51.6 ± 10.2 years and a BMI of 25.2 ± 3.4 kg/m², while the ALNM-negative group had a mean age of 48.9 ± 9.7 years and a BMI of 24.7 ± 3.3 kg/m². Among the enrolled patients, 114 (52.3%) were premenopausal and 104 (47.7%) postmenopausal.

**Table 1:** Demographics and HRV of BC patients.

Variables	Overall (N = 218)	ALNM-positive (N = 142)	ALNM-negative (N = 76)	*P* value
Age (year)	50.6 ± 10.1	51.6 ± 10.2	48.9 ± 9.7	0.026
Height (cm)	159.5 ± 5.3	159.0 ± 5.5	160.3 ± 4.9	0.108
Weight (kg)	63.6 ± 8.9	63.7 ± 8.9	63.4 ± 8.9	0.805
BMI (kg/m2)	25.0 ± 3.4	25.2 ± 3.4	24.7 ± 3.3	0.258
MeanHR (bpm)	72.7 ± 9.1	72.3 ± 8.8	73.6 ± 9.7	0.396
Menopausal state				<0.01
No (n, %)	114 (52.3)	63 (44.4)	51 (67.1)	
Yes (n, %)	104 (47.7)	79 (55.6)	25 (32.9)	
ER state				0.747
No (n, %)	69 (31.7)	46 (32.4)	23 (30.3)	
Yes (n, %)	149 (68.3)	96 (67.6)	53 (69.7)	
PR state				0.040
No (n, %)	101 (46.3)	73 (51.4)	28 (36.8)	
Yes (n, %)	117 (53.7)	69 (48.6)	48 (63.2)	
HER2 state				0.060
No (n, %)	132 (61.7)	80 (57.1)	52 (70.3)	
Yes (n, %)	82 (38.3)	60 (42.9)	22 (29.7)	
Ki-67 state				<0.01
No (n, %)	66 (30.4)	30 (21.3)	36 (47.4)	
Yes (n, %)	151 (69.6)	111 (78.7)	40 (52.6)	

As shown in **Table 2**, conventional HRV metrics for both raw and detrended data showed no significant differences between the ALNM-positive and ALNM-negative groups. **Table 3** summarizes the distribution characteristics of Poincaré plot metrics derived from both raw and detrended data. Analysis of raw data revealed normal distributions for GDE and GDR. Subsequently, Student’s *t* test revealed significant intergroup differences in these metrics: GDE (*p* = 0.023, Cohen’s *d* = 0.30) and GDR (*p* = 0.030, Cohen’s *d* = 0.31). Notably, the observed group differences in GDE and GDR disappeared following smoothness priors detrending. For the detrended AI, Cohen’s *d* was 0.40 (95% CI: 0.12-0.68).

**Table 2:** Comparison of traditional metrics for raw and detrended data between ALNM+ and ALNM-.

Variables	ALNM - positive (N = 142)	ALNM - negative (N = 76)	*P* value
Raw			
SDNN (ms)	26.8 (20.0, 35.7)	28.8 (20.1, 35.8)	0.492
RMSSD (ms)	16.8 (11.4, 23.9)	20.2 (12.0, 30.1)	0.119
LF (ms2)	113 (51, 206)	108 (70, 229)	0.506
HF (ms2)	114 (41, 236)	135 (68, 351)	0.232
LF/HF	0.944 (0.568, 2.133)	1.103 (0.468, 2.031)	0.818
ApEn	1.131 (1.057, 1.188)	1.128 (1.068, 1.189)	0.975
SampEn	2.160 ± 0.327	2.216 ± 0.373	0.250
Smoothness priors detrending method			
SDNN (ms)	14.8 (11.3, 21.4)	17.1 (11.7, 25.4)	0.133
RMSSD (ms)	16.7 (11.3, 23.9)	20.0 (11.9, 29.9)	0.121
LF (ms2)	100 (46, 186)	101 (64, 213)	0.420
HF (ms2)	114 (41, 236)	135 (68, 351)	0.230
LF/HF	0.843 (0.521, 1.937)	0.989(0.432, 1.807)	0.875
ApEn	1.143 ± 0.083	1.146 ± 0.097	0.835
SampEn	2.461 (2.308, 2.646)	2.479 (2.337, 2.709)	0.354

**Table 3:** Comparison of Poincaré plot graphical metrics for raw and detrended data between ALNM+ and ALNM-.

Variables	ALNM-positive (N = 142)	ALNM-negative (N = 76)	*P* value
Raw			
SD1 (ms)	11.9 (8.1, 17.0)	14.3 (8.5, 21.3)	0.120
SD2 (ms)	35.1 (26.8, 47.6)	37.7 (27.0, 46.1)	0.705
SD1/SD2	0.3 (0.2, 0.5)	0.4 (0.3, 0.5)	0.080
SI^*^	0.323 (0.113, 0.619)	0.303 (0.180, 0.561)	0.871
PI^*^	2.803 (1.360, 4.307)	2.967 (1.429, 4.481)	0.893
GI^*^	0.322 (0.114, 0.630)	0.300 (0.179, 0.547)	0.861
AI^*^	0.327 (0.116, 0.627)	0.300 (0.178, 0.533)	0.829
GDR	0.016 ± 0.002	0.017 ± 0.002	0.030
GDE	5.773 (5.695, 5.840)	5.812 (5.734, 5.867)	0.023
Smoothness priors detrending method			
SD1 (ms)	11.8 (8.0, 16.9)	14.1 (8.4, 21.2)	0.121
SD2 (ms)	17.2 (12.3, 24.3)	19.7 (13.3, 27.7)	0.193
SD1/SD2	0.6 (0.6, 0.8)	0.7 (0.6, 0.8)	0.657
SI^*^	3.335 (1.585, 6.142)	4.239 (2.120, 6.463)	0.133
PI^*^	2.061 (0.944, 3.369)	1.994 (0.737, 3.872)	0.779
GI^*^	0.107 (0.054, 0.213)	0.150 (0.076, 0.220)	0.062
AI^*^	2.832 (1.331, 4.819)	3.688 (2.176, 6.537)	0.006
GDR	0.017 ± 0.002	0.018 ± 0.002	0.213
GDE	5.824 ± 0.116	5.845 ± 0.121	0.214

*SI, PI, GI, AI values denote absolute deviations calculated as |measurement - 50|.

As shown in **Table 4**, after adjusting for age, menopausal status, PR expression, and Ki-67 index, the detrended AI remained significantly different between the ALNM-positive and ALNM-negative groups (adjusted *p* = 0.024, partial η² = 0.024). In contrast, raw GDR and GDE were no longer statistically significant after adjustment (adjusted *p* = 0.152 and 0.193, respectively). These findings suggest that the detrended AI reflects a metastasis-specific autonomic signature that is independent of demographic and tumor-related confounders.

**Table 4:** ANCOVA for Poincaré plot metrics, adjusted for age, menopausal status, PR expression, and Ki-67 index.

Variables	Data preprocessing	*P* value	Adjusted *p* value	Partial η²
AI^*^	Detrending	0.006	0.024	0.024
GDR	Raw	0.030	0.152	0.010
GDE	Raw	0.023	0.193	0.008

Adjusted *p* values were rigorously adjusted using an ANCOVA model. To comprehensively control for both systemic physiological baseline and tumor-specific biological aggressiveness, the following covariates were included: Age, Menopausal status, PR expression, and Ki-67 proliferation index. An adjusted *p* < 0.05 indicates that the metric is an independent predictor of ALNM,Partial η² is the adjusted effect size.

**Figure 8:**
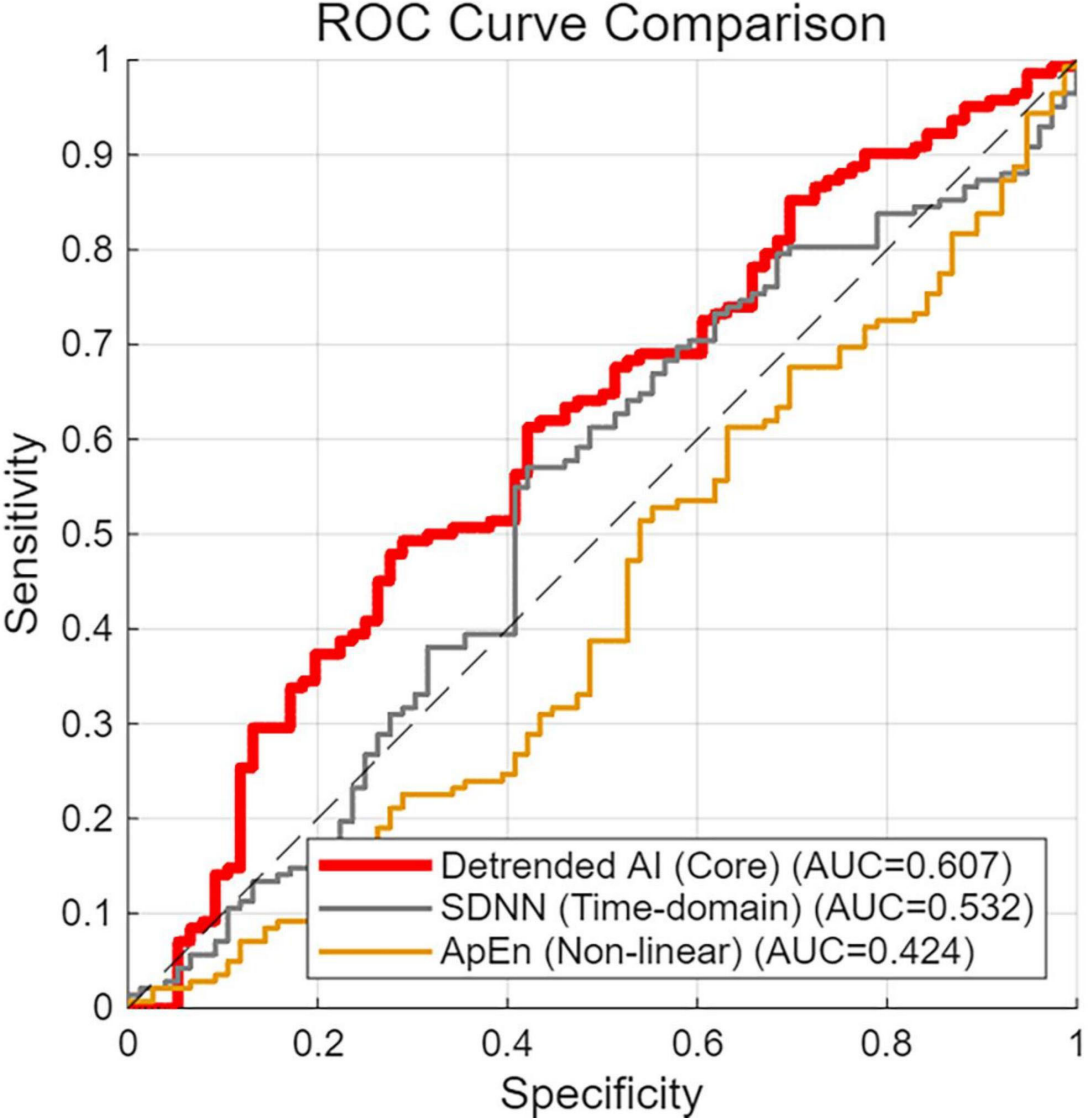
ROC comparison for ALNM prediction after 10-fold cross-validation. The red curve represents the core novel metric, detrended AI; the gray curve represents the conventional time-domain HRV metric, standard deviation of normal-to-normal intervals; the yellow curve represents the conventional non-linear HRV metric, approximate entropy. The diagonal dashed line indicates the performance of a random guess (AUC = 0.5). The detrended AI showed a discriminative ability that was modest but notably better than that of conventional HRV metrics for ALNM prediction.

ROC analysis was conducted to evaluate the discriminative performance of the detrended AI. The original AUC was 0.612. To assess robustness against overfitting, a 10-fold cross-validation was then performed. The cross validated AUC was 0.607, with a sensitivity of 86.84% and a specificity of 38.73%, which was highly consistent with the original AUC of 0.612, suggesting that the predictive performance was not attributable to overfitting to the specific cohort. In comparison, conventional HRV metrics (e.g., SDNN) showed an AUC of 0.532 and non-linear HRV metrics (e.g., ApEn) showed an AUC of 0.424, indicating limited discriminative ability (**Figure 8**).

## 4 Discussion

This study compared the efficacy of conventional HRV metrics and Poincaré plot graphical metrics in assessing ANS function in BC patients with ALNM under both raw and detrended data conditions. The results showed that Poincaré plots graphical metrics—specifically AI from detrended data, along with GDR and GDE from raw data were significantly different discriminated ALNM status (*p* < 0.05), with markedly reduced values in the metastasis group. In contrast, conventional HRV metrics (SDNN, RMSSD, LF, HF, LF/HF, ApEn, SampEn) and standard Poincaré plot graphical metrics (SD1, SD2, SD1/SD2) showed relatively consistent trends across both subgroups. These findings suggest a potential methodological advantage of RRI-based graphical analysis in capturing ALNM-associated ANS alterations.

The contrast in statistical significance between the conventional metrics (**Table 2**) and the Poincaré plot metrics (**Table 3**) offers pathophysiological insights. As shown in **Table 2**, all conventional time- and frequency-domain metrics failed to reach statistical significance (*p* > 0.05). Statistically, this signifies that the global magnitude of sympathovagal balance is not overtly or specifically disrupted by early axillary lymphatic spread. Physiologically, this suggests that conventional linear analyzes are likely obscured by the general metabolic noise of the primary tumor burden. Conversely, the statistically significant *p*-values observed in **Table 3** specifically for the detrended AI (*p* = 0.006) suggest the presence of a robust, non-linear structural alteration in autonomic beat-to-beat dynamics. This statistical divergence suggests that ALNM does not merely induce a global autonomic depression, but rather triggers a highly specific, localized neuro-inflammatory spatial asymmetry that may be better captured by advanced non-linear graphical algorithms.

For raw RRI data analysis, the ALNM-positive group exhibited significantly reduced GDR and GDE values, suggesting decreased dispersion density (GDR↓) and diminished complexity (GDE↓) of RRI distributions in Poincaré plots among metastasis group patients. This phenomenon may represent a graphical manifestation of reduced ANS regulatory flexibility: during metastatic progression, sustained sympathetic activation (Le et al., 2016; An et al., 2021) coupled with vagal suppression (Renz et al., 2018) may collectively contribute to HRV “stereotypization” (De Couck and Gidron, 2013), thereby potentially impairing the dynamic regulatory capacity of cardiac rhythm in response to physiological challenges (Kim et al., 2010; Arab et al., 2016; Monje, 2017).

Analysis of detrended data revealed a significantly higher AI in the ALNM-negative group compared to the ALNM-positive group (significantly lower in ALNM-positive), a pattern that was reversed in raw data analysis. This finding may reflect diminished parasympathetic regulation in the ALNM-positive group (Erin et al., 2008; Erin et al., 2012), suggesting that AI calculated from raw data may be susceptible to low-frequency noise contamination, potentially leading to artificial inflation. Following removal of low-frequency noise through detrending, AI appeared to more accurately detect ANS dysregulation. This could account for the significantly higher AI values observed in detrended data compared to raw data. Simultaneously, for the non-metastatic group, detrending may have removed some low-frequency physiological components, thereby rendering its signal distribution more closely resembling that of the metastatic group. In the ALNM-positive group, the significant alteration in AI manifested graphically as a reduction in spatial asymmetry of acceleration/deceleration processes, suggesting possible autonomic imbalance dominated by vagal inhibition (Tarvainen et al., 2014). This finding appears consistent with the impairment of the cholinergic anti-inflammatory pathway within the tumor microenvironment (Tracey, 2002; Rosas-Ballina et al., 2011). Such dysfunctional neuroregulation may indirectly contribute to tumor metastasis by exacerbating inflammatory responses, promoting angiogenesis, and inducing immune suppression (Tracey, 2009). In contrast, conventional non-linear entropy metrics (e.g., ApEn, SampEn), while capable of quantifying signal complexity, lack intuitive graphical depiction of RRI dynamics in phase space and fail to characterize spatial structural features (Woo et al., 1992); consequently, they may exhibit reduced sensitivity in detecting autonomic functional alterations associated with ALNM (Tulppo et al., 1996). Conversely, conventional time-frequency metrics, being reliant on steady-state signals, are inadequate for quantifying spatial heterogeneity in transient dynamic ECG signals. Moreover, even with detrending applied, they remain insufficient for capturing direction-specific pathological features during short-term dynamic fluctuations. Consequently, these metrics showed limited predictive performance in this study (Huikuri et al., 2000; Billman, 2013).

A critical physiological question is whether these observed autonomic alterations reflect the total macroscopic tumor burden or a systemic inflammatory response specifically driven by the metastatic cascade. While an increasing primary tumor mass may induce a global, nonspecific depression of overall autonomic tone (often reflected by diminished linear metrics such as SDNN), the process of ALNM may involve more specific neuro-immune crosstalk. Current evidence suggests that metastasis can be facilitated by localized sympathetic hyperactivation, which may drive lymphangiogenesis (Sloan et al., 2010) and trigger a systemic inflammatory cytokine cascade (e.g., IL-6, TNF-α) that centrally modulates vagal efferent activity (Pavlov and Tracey, 2012). Based on these findings, this present study suggests that it is this metastasis-specific, dynamic neuro-inflammatory interplay, rather than solely the metabolic burden of tumor mass that may contribute to the subtle spatial asymmetry in beat-to-beat variability captured by the detrended AI. This interpretation is supported by our multivariable analysis, in which the detrended AI remained significantly different between groups after adjusting for the Ki-67 proliferation index, a well-established proxy for tumor proliferative activity and disease aggressiveness. However, given the exploratory nature of this study and the absence of direct measurements of inflammatory cytokines or sympathetic nerve activity, this mechanistic interpretation remains speculative. Future studies incorporating direct biomarkers of neuro-immune interaction are needed to elucidate the underlying pathways.

A notable methodological observation in this study is that while raw GDR and GDE exhibited group differences, these differences were no longer apparent following the detrending process. This phenomenon may be explained by the mathematical and physiological nature of these specific metrics. GDR and GDE quantify the macroscopic spatial dispersion of RR intervals across the Poincaré grid. As such, they may be more susceptible to low-frequency physiological fluctuations, such as respiratory sinus arrhythmia, slow autonomic baseline drifts, and hormonal variations. Detrending may act as a high-pass filter, removing some of these non-specific, macroscopic trends. The fact that GDR and GDE differences were not retained after detrending might suggest that their initial significance was influenced by systemic physiological noise rather than reflecting the metastatic process itself. In contrast, the detrended AI focuses on microscopic, beat-to-beat spatial asymmetry. By filtering out macro-level geometric distortion, detrending may help reveal a more subtle non-linear autonomic signal, which in this cohort appeared to be associated with ALNM status. This could explain why AI emerged as a relatively robust predictor in our multivariable analysis.

Poincaré plots were adopted for graphical analysis in this study. This approach offers: (a) computational simplicity, (b) intuitive result visualization, (c) effective characterization of short- and long-term RRI variability and correlations, and (d) quantifiable assessment of plot asymmetry and distribution complexity (Woo et al., 1992). The recurrence plot (RP), another significant non-linear graphical analysis technique, generates a two-dimensional binary image through phase space reconstruction. This is achieved by quantifying the recurrence patterns of state vectors within a time series. RP effectively characterizes critical dynamic features including periodicity, non-stationary behavior, and transient dynamics (Acharya et al., 2005). However, while RP effectively captures dynamic stability, their binary representations and derived metrics lack the capacity to directly associate with specific ANS pathway dysregulations within the tumor microenvironment. Additionally, RP exhibits higher computational complexity than conventional time-domain analyzes. This study employs Poincaré plots as the primary analytical framework. This approach was prioritized because it reconstructs complex ANS dynamics into geometrically interpretable patterns within phase space, while providing relatively clear associations between quantifiable features and underlying tumor pathological mechanisms. Future investigations may explore either RP or integrated analytical frameworks combining Poincaré plots with RP. Such multimodal approaches may more comprehensively characterize dynamic signatures of tumor-related ANS dysregulation within HRV profiles.

This study has several limitations that should be explicitly acknowledged. First, the data originated from a single center with a moderate sample size and an inherent class imbalance between ALNM-positive and ALNM-negative groups. Future multi-center studies with expanded cohorts are essential to validate the generalizability of these findings. Second, and importantly, all participants were already aware of their breast cancer diagnosis at the time of ECG recording. This awareness inevitably induces varying degrees of psychological stress, anxiety, and heightened sympathetic activation, all of which are potent confounders of autonomic tone. Consequently, the observed HRV differences may partially reflect the profound psychological burden of a cancer diagnosis in addition to the physiological impact of metastatic spread. Future investigations should incorporate standardized psychological psychometric assessments to strictly adjust for this confounder. Finally, the observation that detrending eliminated group differences in raw GDR and GDE suggests that the choice of detrending algorithms and grid parameters may influence spatial metrics. Further methodological studies are needed to establish optimal, standardized preprocessing strategies for HRV analysis in oncological settings.

To overcome these current limitations, it is appropriate to frame the present work as an exploratory, proof-of-concept investigation. Future research should transition from this initial exploration toward more rigorous, large-scale, multi-center prospective validation. Specifically, subsequent study designs could benefit from incorporating standardized psychometric evaluations alongside concurrent neuroendocrine biomarker profiling to better distinguish ALNM-specific physiological signals from the psychological stress associated with a cancer diagnosis. In addition, the integration of advanced machine learning frameworks may facilitate the development of adaptive signal processing algorithms, enabling automated, patient-specific optimization of detrending parameters and grid resolutions. Such comprehensive, multidisciplinary approaches could help translate these exploratory spatial HRV metrics into more robust, potentially clinically useful adjunctive tools in the field of onco-cardiology.

## 5 Conclusions

In conclusion, this exploratory study suggests that specific non-linear spatial asymmetry in heart rate variability, particularly the detrended AI, may serve as a potential non-invasive indicator of autonomic dysregulation associated with ALNM in breast cancer patients. While conventional linear HRV metrics failed to demonstrate predictive value, the detrended AI appeared to capture a subtle autonomic signature that may be related to ALNM, independent of total tumor burden and baseline confounders. However, given the modest discriminatory ability (AUC = 0.612) and the single-center nature of this study, these findings must be interpreted with caution. The proposed Poincaré metrics are not currently intended to replace standard clinical diagnostics, but rather to offer a novel, exploratory pathophysiological perspective on the neuro-neoplastic microenvironment. Large-scale, multi-center prospective studies incorporating psychological adjustments are needed to validate the clinical generalizability and utility of these indices.

## Data Availability

The raw data supporting the conclusions of this article will be made available by the authors, without undue reservation.
